# Synthesis and Properties of Acyclic Ammonium-based Ionic Liquids with Allyl Substituents as Electrolytes

**DOI:** 10.3390/molecules14051840

**Published:** 2009-05-15

**Authors:** Taeeun Yim, Chang Young Choi, Junyoung Mun, Seung M. Oh, Young Gyu Kim

**Affiliations:** Department of Chemical and Biological Engineering, Research Center for Energy Conversion & Storage, Seoul National University, Seoul 151-744, Korea; E-mails: yte0102@snu.ac.kr (T.Y.), max923@snu.ac.kr (C-Y.C.), river7@snu.ac.kr (J.M.)

**Keywords:** ionic liquid, ammonium, bis(trifluoromethanesulfonyl)imide (TFSI), allyl substituent, battery electrolyte

## Abstract

Several new acyclic ammonium-TFSI ionic liquids with an allyl substituent(s) were synthesized and their physicochemical and electrochemical properties were characterized. [AAMM]Am-TFSI (**3**) with two allyl groups showed the widest electrochemical stability window (5.9 V) among the ammonium-based ILs reported to date because of the increment of both the anodic and cathodic limits. The charge-discharge performance of a LiCoO_2_-based half-cell containing [AAMM]Am-TFSI as an electrolyte was better in cycleability (the capacity retention ratio: 99% after 20 cycles) than that of the cell with the corresponding partially saturated analogue, [AMMP]Am-TFSI (**2**) (the capacity retention ratio: 92% after 20 cycles).

## 1. Introduction

Ionic liquids (ILs) are salts that exist as liquid at low temperature although they are composed of ionic species, cations and anions. Owing to the ionic nature in bonding, they have unique properties such as high ionic conductivity, non-volatility, and non-flammability as well as a wide liquid range and wide electrochemical stability window [1]. Furthermore, the designability of ILs makes themselves an attractive alternative to the conventional organic electrolytes and solvent systems [2,3,4,5].

Recently, we have reported the physicochemical and electrochemical properties of several imidazolium-based ILs with unsaturated substituents [6,7]. Most of them exhibit a lower viscosity, higher ionic conductivity, and wider voltage window than their saturated analogues. However, the imidazolium-based ILs do not seem appropriate as lithium battery electrolytes because they are electrochemically unstable particularly under cathodic polarization, which is mainly due to the acidic proton at the C-2 position of the imidazolium ring. One way to improve the cathodic stability of ILs would be to change the cation from imidazoliums to ammoniums that have no acidic protons [8].

As part of our ongoing study, acyclic ammonium ions have been selected as cations with allyl substituents and the results are reported here [9]. The allylic substituent was expected to increase both the physicochemical properties and anodic stability of the target ILs as reported in our work [6,7]. We have also prepared the allyl-substituted acyclic ammoniums with an heteroatom substituent such as an oxygen that is known to enhance the physicochemical properties of the acyclic ammonium ILs [4,10].

## 2. Results and Discussion

### 2.1. Synthesis and characterization of products

**Scheme 1:**
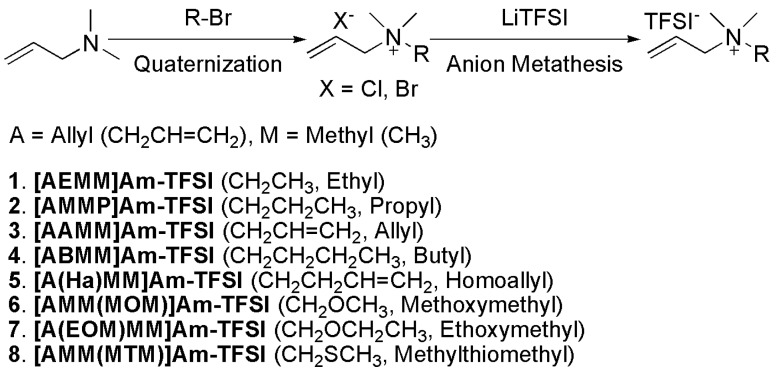
Preparation of allyl-substituted ammonium-TFSI ILs.

The desired quaternary ammonium ILs were synthesized effectively according to the reported procedure [8], i.e., a quaternization reaction of commercially available allyldimethylamine followed by an anion metathesis (Scheme 1). The structures of products were confirmed by their ^1^H- and ^13^C-NMR spectra, and elemental analyses.

### 2.2. Thermal and physical properties

**Figure 1:**
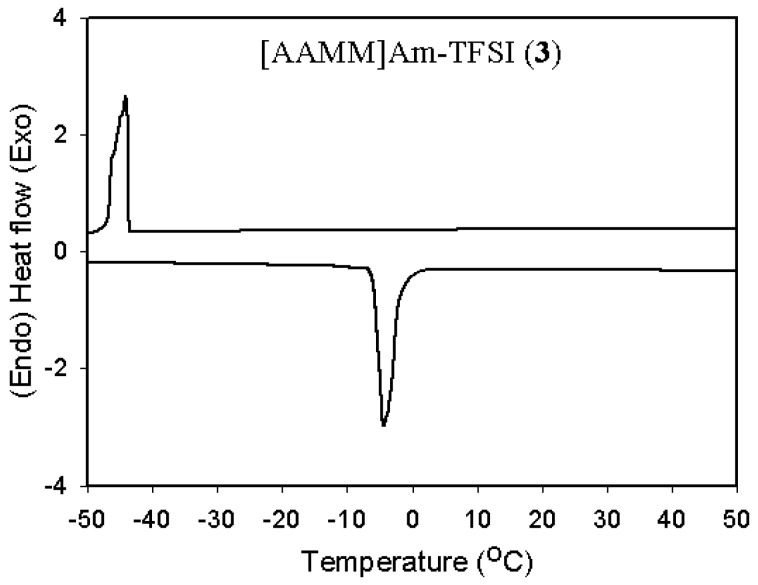
The DSC chart of [AAMM]Am-TFSI (**3**).

Table 1 shows the thermal and physicochemical properties of the ILs prepared in this work; melting point (*T*_m_), crystallization point (*T*_c_), decomposition temperature (*T*_d_), viscosity (*η*), and ionic conductivity (*σ*) (see Scheme 1 for their structures and abbreviations). The DSC chart of [AAMM]Am-TFSI (**3**) is also shown in Figure 1. All of the allyl-substituted ammonium-TFSI ILs in the present study are liquid at room temperature. The ILs with heteroatom-containing substituents are thermally less stable than their alkyl derivatives (entries 1-5 *vs.* 6-8), probably owing to the reactive nature of an acetal functionality. In particular, IL **8** with a sulfur-substituted substituent is noticeably less stable (entry 8). We have also measured the thermal stability of some ILs (**2**, **3** and **6)** with a slower heating rate (5 ^o^C min^-1^); **2** (281 ^o^C), **3** (286 ^o^C), and **6** (234 ^o^C). Their *T*_d_ values at the slower heating rate are decreased by about 8-34 ^o^C compared to those measured with the higher rate (10 ^o^C min^-1^) (entries 2, 3 and 6). However, all of the ILs in the present study are still more stable than conventional organic electrolytes such as ethylene carbonate and propylene carbonate. 

Regarding viscosity, the ILs with an additional allylic substituent show lower values than those of the corresponding saturated analogues (entries 2 *vs.* 3 and 4 *vs.* 5), probably due to the more or less planar structure of the allyl group. The similar trend was observed in previous reports [6,7,8]. When the methylene (CH_2_) group is replaced with an oxygen atom, the decrease in viscosity is significant (entries 2 *vs.* 6 and 4 *vs.* 7) as reported in the literature [4,10]. Concerning conductivity, IL **6** with the lowest viscosity (47 cP, entry 6) shows the highest ionic conductivity (3.9 mS cm^-1^) among the ILs prepared. On the contrary, the substitution with a sulfur atom results in an increase of the viscosity and the lowest conductivity is observed (entry 8). Introduction of an allyl group seems to have a certain positive effect on the conductivity of IL **3** as well (entries 2 *vs.* 3), whereas the decreased conductivity of IL **5** with a homoallyl group is shown compared to its saturated analogue IL **4** (entries 4 *vs.* 5). However, these ammonium-TFSI ILs still have the inferior physicochemical properties to those reported in the literature for [EM]Im-TFSI (34 cP, 9.2 mS cm^-1^) [10]. The ionic conductivities of some ILs, **2** (0.77 mS cm^-1^), **3** (1.1 mS cm^-1^) and **6** (1.5 mS cm^-1^), with 1.0 M LiTFSI are lower than those without the LiTFSI salt (entries 2, 3 and 6). 

### 2.3. Electrochemical properties

Electrochemical stability of the ILs was determined by linear sweep voltammetry and the results are shown in Table 2. As expected, the cathodic stability of all the ammonium-based ILs is much improved compared to that of the reported imidazolium-based ILs [6,7]. To our excitement, IL **3** shows the highest anodic stability (3.4 V, entry 3), resulting in the widest electrochemical stability window of 5.9 V. The similar enhancement in anodic stability by an allylic substituent was observed with the imidazolium-BF_4_ ILs [6,7].

Because similar anodic stability is to be expected for the ILs having the same anion [11], the additional allylic group should be responsible for its higher anodic limit. The reason for this is not clear at the present, but a certain type of stabilization seems at work. In the literature, allyl ethyl carbonate was claimed to form a good quality SEI (solid electrolyte interphase) film on a graphite negative electrode [12]. Although this allyl effect was not clearly shown with the cyclic ammonium-TFSI ILs, it was evident with the imidazolium-TFSI ILs [8]. The anodic limit of [AA]Im-TFSI was close to +3.0 V, whereas that of [AM]Im-TFSI was about +2.0 V (*vs.* Ag/Ag^+^). It seems also supported by the improved stability of IL **5**, albeit small, at its anodic limit (entry 5). 

**Figure 2:**
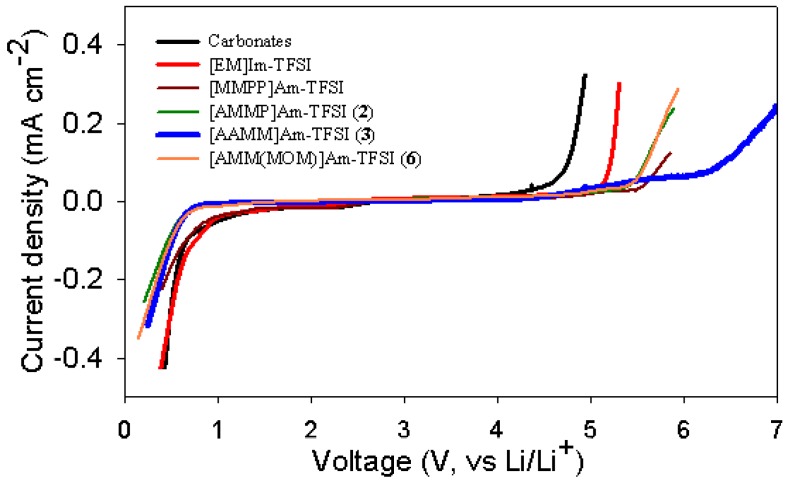
Electrochemical stability windows of the ILs with 1.0 M LiTFSI.

We have also tested the electrochemical stability of the ILs together with a lithium salt to demonstrate the feasibility of their use as an electrolyte for lithium ion batteries (Table 3). Thus, both ILs **3** (entry 5) and **6** (entry 6) were measured with 1.0 M LiTFSI and their electrochemical windows were compared with commercially available carbonates (entry 1), [EM]Im-TFSI (entry 2), and their fully (entry 3) or partially saturated analogues (entry 4). These data are also presented in Figure 2.

It is apparent that the ammonium-based ILs show enhanced cathodic stability compared to [EM]Im-TFSI because of the absence of an acidic proton in the ammonium cations (entries 2 *vs.* 3-6). It is also interesting to note that the ammonium ILs with at least one allyl group show wider cathodic windows than their fully saturated analogue (entries 3 *vs.* 4-6). Although IL **3** with two allyl groups shows lesser cathodic stability than IL **2** with one allyl group, the enhancement of the anodic stability with IL **3** is remarkable, resulting in the widest electrochemical window among the ammonium-based ILs reported to date in the literature [10,13]. The similar behavior with the pyrrolidinium-based ILs was noted previously [8]. Thus, the allyl group could be a good alternative to a saturated alkyl group of ammonium-based ILs to increase the width of their electrochemical windows. 

**Figure 3:**
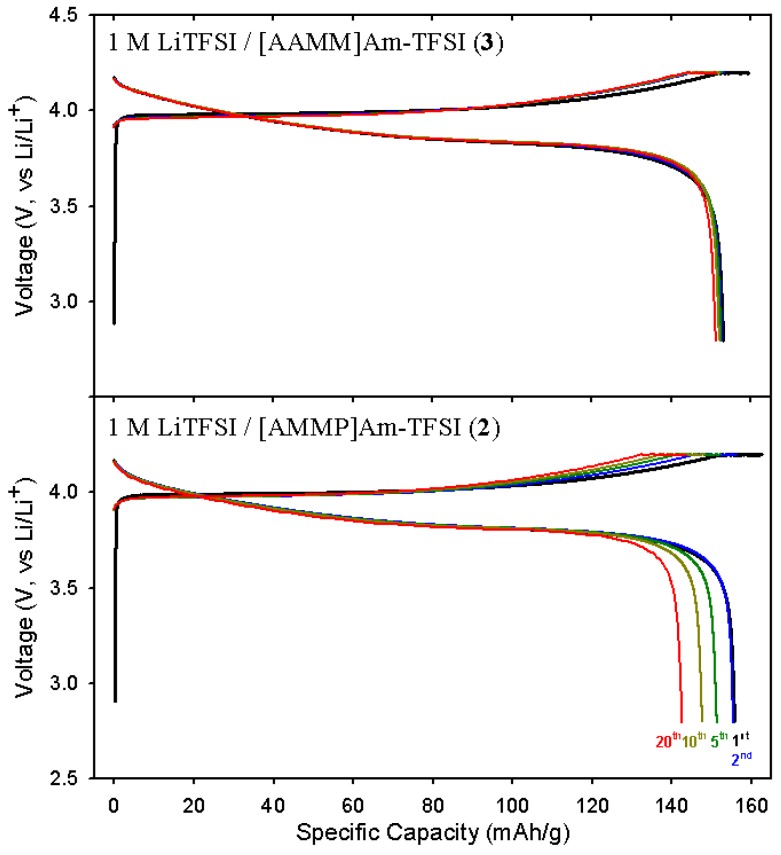
The charge–discharge voltage profiles of Li/1.0 M LiTFSI in ILs/LiCoO_2_ cells.

Among the ILs prepared in the present study, the cell performance of [AAMM]Am-TFSI with the improved electrochemical stability window was tested, and its charge/discharge voltage profile is compared to that of a partially saturated analogue, [AMMP]Am-TFSI, in Figure 3. 

**Figure 4:**
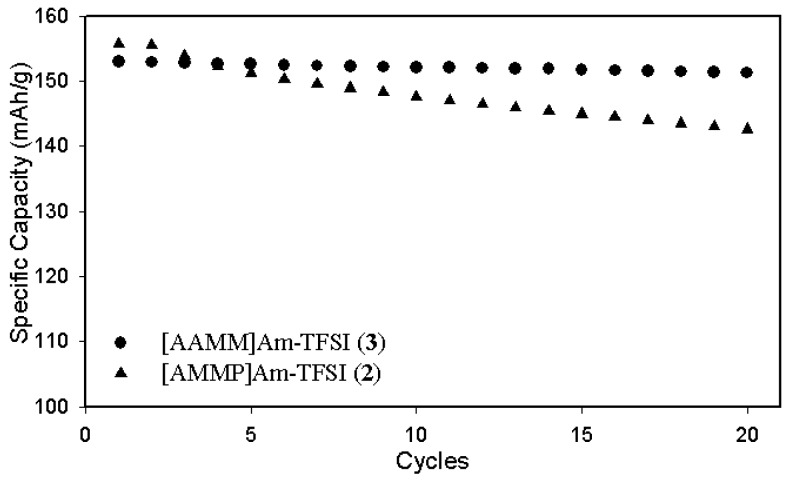
Dependence of the discharge capacities of the cell with the ILs on the cycle number.

The dependence of its discharge capacities upon the cycle number is shown in Figure 4, too. The data given in Figure 3 and Figure 4 indicate clearly that the two allyl groups have a positive effect enhancing the cycleability of the cell. [AAMM]Am-TFSI with two allyl groups showed a better cycleability after the 20^th^ cycle than that of [AMMP]Am-TFSI containing one allyl group. 

Compared to [AMMP]Am-TFSI (the discharge capacity: 143 mAh g^-1^, the capacity retention ratio: 92%), the discharge capacity of the half-cell using [AAMM]Am-TFSI as an electrolyte was highly maintained after 20 cycles (the discharge capacity: 151 mAh g^-1^, the capacity retention ratio: 99%). For the present, we suppose that a certain type of an SEI film on the electrodes formed by the allyl groups of [AAMM]Am-TFSI would contribute the positive effect on the cycling efficiency during the charge-discharge processes. An effort to obtain some definite proof for the formation of the SEI film on the electrodes is now under way.

## 3. Conclusions

Several new allyl-substituted ammonium-TFSI ILs were synthesized and characterized [14]. All of them were liquid at room temperature and stable up to 220 ^o^C. Although the ether-containing ILs were thermally less stable, they showed much improved viscosity and conductivity. The sulfur-containing IL showed poor physical properties. The allyl-containing ammonium-based ILs **2**, **3** and **6** prepared in the present study showed much enhanced cathodic stability compared to both the imidazolium-based ILs and the commercially used carbonates. It seemed that the presence of an allyl group(s) on the acyclic ammonium-based ILs had some positive effect on the electrochemical stability, as well as their physicochemical properties. Particularly, IL **3** with two allyl groups showed the widest electrochemical window (5.92 V) with 1.0 M LiTFSI among the ammonium-based ILs reported to date in the literature [10,13]. It also displayed an improved cycleability and the higher cycling efficiency, probably because of the increment in the anodic stability. Based on the above interesting results, we hope that the ILs with the enhanced cathodic and anodic stability would find many applications as an electrolyte in lithium secondary batteries and capacitors.

## 4. Experimental

### 4.1. General

^1^H- and ^13^C-NMR spectra were recorded with a JEOL JNM LA-300 spectrometer (300 MHz for ^1^H- NMR and 75 MHz for ^13^C-NMR) using DMSO as solvent. The ^1^H-NMR data are reported in ppm (*δ*) from the internal standard (TMS, 0.0 ppm) as follows: chemical shift (multiplicity, coupling constant in Hz, integration), and the ^13^C-NMR data in ppm (*δ*) from the internal standard (TMS, 0.0 ppm). Viscosity (*η*) was measured on a Brookfield DV-II+ cone/plate viscometer. Crystallization points (*T*_c_) and melting points (*T*_m_) were determined by using a TA Instruments Differential Scanning Calorimeter (DSC) Q1000 under a N_2_ atmosphere. Thermograms were acquired during heating from -50 ^o^C to 100 ^o^C scans at a heating rate of 10 ^o^C min^−1^ after cooling to -50 ^o^C scans at a cooling rate of 10 ^o^C min^−1^. Thermal decomposition temperatures (*T*_d_) were determined by using SDT Q600 and Mettler TG 50 TA instruments. Heating rate and terminal temperature were set at 10 ^o^C min^−1^ and 500 ^o^C, respectively, unless stated otherwise. Thermal decomposition temperature was recorded at 1% weight loss temperature. Ionic conductivity (*σ*) was determined by the complex impedance measurements between symmetric platinum electrodes, using a CHI660A electrochemical workstation in the frequency range from 1 Hz to 100 kHz. The cell constant of the cell was 1.6 cm^−1^, determined with a 1.0 M KCl aqueous solution. Linear sweep voltammetry of neat state ILs was performed with a CHI660A electrochemical workstation for the electrochemical stability window measurement at a scan rate of 10 mV s^−1^ by using a glassy carbon electrode (7.07 × 10^−2^ cm^2^) as a working electrode. The working electrode was polished before every measurement. Platinum electrode was used as a counter electrode and silver wire as a quasi-reference electrode. The potential of silver wire reference was calibrated by a ferrocene redox couple. In this work, the cathodic and anodic limits were arbitrarily defined as the potentials at which the current density reached 1 mA cm^−2^ (without 1.0 M LiTFSI) or 0.1 mA cm^−2^ (with 1.0 M LiTFSI).

For the preparation of a LiCoO_2_-containing composite electrode, a slurry mixture of LiCoO_2_ powder, Super-P (as a carbon additive for conductivity enhancement) and poly(vinylidenefluoride) (PVdF, as a binder) (95:3:2 wt% ratio) was coated on a piece of an Al current collector, which was followed by drying at 120 ^o^C. Coin-type half-cells were fabricated with the composite electrode, Li foil as a counter and reference electrode, and poly(propylene) as a separator. Galvanostatic discharge-charge cycling was made using a Toscat-3100 cycler and the charge-discharge tests on the cells were performed at 4.3-2.8 V of cut-off voltage. The current density is 0.1 C at 25 ^o^C and the cells were fully charged and discharged by constant current/constant voltage (CCCV) and constant current (CC) modes, respectively. 

### 4.2. General procedure for the preparation of acyclic ammonium halides

To a stirred solution of allyldimethylamine (100.0 mmol) in acetonitrile (80 mL) was added alkyl halide (120.0 mmol) dropwise at 0 ^o^C. The reaction mixture was stirred for 24 h at 30 ^o^C. Completion of the reaction was confirmed by ^1^H- and ^13^C-NMR spectra. Removal of the solvent under reduced pressure afforded crude tetraalkylammonium halide. The crude product was purified by salting out in a mixture of acetonitrile and ethyl acetate.

*[AEMM]Am-Br*: Quantitative yield; ^1^H-NMR δ 1.25 (t, *J* = 7.3, 3H), 2.98 (s, 6H), 3.33 (q, *J* = 7.3, 2H), 3.97 (d, *J* = 7.1, 2H), 5.62 (d, *J* = 9.0, 1H), 5.64 (d, *J* = 17.6, 1H), 5.98-6.12 (m, 1H); ^13^C-NMR δ 7.8, 49.0 (t, *J*_C-N_ = 3.8), 58.5 (t, *J*_C-N_ = 3.1), 64.5 (t, *J*_C-N_ = 2.5), 126.0, 127.3.

*[AMMP]Am-Br*: Quantitative yield; ^1^H-NMR δ 0.89 (t, *J* = 7.3, 3H), 1.64-1.77 (m, 2H), 2.98 (s, 6H), 3.15-3.21 (m, 2H), 3.94 (d, *J* = 7.3, 2H), 5.62 (d, *J* = 14.5, 1H), 5.62 (d, *J* = 11.5, 1H), 5.98-6.12 (m, 1H); ^13^C-NMR δ 10.4, 15.2, 49.6 (t, *J*_C-N_ = 3.7), 64.3, 65.1, 125.9, 127.3.

*[AAMM]Am-Br*: Quantitative yield; ^1^H-NMR δ 2.96 (s, 3H), 3.93 (d, *J* = 7.0, 2H), 5.63 (d, *J* = 15.2, 1H), 5.64 (d, *J* = 11.4, 1H), 6.00-6.14 (m, 1H); ^13^C-NMR δ 49.3 (br), 65.1, 125.9, 127.7.

*[ABMM]Am-Br*: Yield 80%; ^1^H-NMR δ 0.93 (t, *J* = 7.1, 3H), 1.30 (sext, *J* = 7.5, 2H), 1.62-1.72 (m, 2H), 2.99 (s, 6H), 3.20-3.26 (m, 2H), 3.96 (d, *J* = 7.1, 2H), 5.62 (d, *J* = 11.4, 1H), 5.63 (d, *J* = 15.6, 1H), 5.98-6.12 (m, 1H); ^13^C-NMR δ 13.4, 19.1, 23.5, 49.5 (t, *J*_C-N_ = 3.8), 62.7 (t, *J*_C-N_ = 2.5), 65.0 (t, *J*_C-N_ = 2.5), 125.9, 127.3.

*[A(Ha)MM]Am-Br*: Yield 80%; ^1^H-NMR δ 2.47-2.55 (m, 2H), 3.02 (s, 6H), 3.29-3.35 (m, 2H), 3.98-4.01 (m, 2H), 5.15 (d, *J* = 10.2, 1H), 5.23 (d, *J* = 17.2, 1H), 5.63 (d, *J* = 10.2, 1H), 5.64 (d, *J* = 16.7, 1H), 5.68-5.82 (m, 1H), 6.00-6.14 (m, 1H); ^13^C-NMR δ 26.2, 49.6 (t, *J*_C-N_ = 3.7), 61.6 (t, *J*_C-N_ = 2.5), 65.2 (t, *J*_C-N_ = 3.1), 118.2, 125.9, 127.4, 132.9.

*[AMM(MOM)]Am-Cl*: Quantitative yield; ^1^H-NMR δ 3.00 (s, 6H), 3.63 (s, 3H), 4.01 (d, *J* = 7.3, 2H), 4.71 (s, 2H), 5.61 (d, *J* = 9.2, 1H), 5.63 (dd, *J* = 17.8 and 1.1, 1H), 5.97-6.10 (m, 1H); ^13^C-NMR δ 46.3 (t, *J*_C-N_ = 3.8), 60.7, 62.3, 91.1, 125.5, 127.5.

*[A(EOM)MM]Am-Cl*: Quantitative yield; ^1^H-NMR δ 1.21 (t, *J* = 7.1, 3H), 2.95 (s, 6H), 3.84 (q, *J* = 7.0, 2H), 3.94 (d, *J* = 7.3, 2H), 4.70 (s, 2H), 5.61 (d, *J* = 14.6, 1H), 5.62 (d, *J* = 11.7, 1H), 5.96-6.10 (m, 1H); ^13^C-NMR δ 15.0, 46.2, 62.2, 68.8, 89.7, 125.6, 127.5.

*[AMM(MTM)]Am-Cl*: Quantitative yield; ^1^H-NMR δ 2.49 (s, 3H), 3.15 (s, 6H), 4.17 (d, *J* = 7.1, 2H), 4.87 (s, 2H), 5.70 (d, *J* = 9.3, 1H), 5.72 (d, *J* = 18.3, 1H), 6.07-6.21 (m, 1H); ^13^C-NMR δ 18.0, 48.2, 64.3, 69.4, 125.9, 127.7.

### 4.3. General procedure for anion metathesis

To a solution of tetraalkylammonium halide (100.0 mmol) in distilled water (80 mL) was added lithium bis(trifluoromethanesulfonyl)imide (100.0 mmol). The reaction mixture was stirred for 24 h at 80 ^o^C. After the reaction mixture was cooled to room temperature, distilled water (100 mL) was added to the mixture and the resulting mixture was extracted with dichloromethane (2 x 100 mL). The combined organic layers were washed with fresh distilled water (2 x 50 mL) and then dried over MgSO_4_. The crude organic solution was filtered through neutral aluminum oxide to remove any precipitated lithium salt and the yellowish color. The colorless solution was concentrated by removing the solvent under reduced pressure to afford the desired tetraalkylammonium bis(trifluoromethanesulfonyl)imide. The water content of the ILs prepared in the present study was less than 0.005 wt%, measured by Karl-Fischer titration.

*Allyldimethylethylammonium bis(trifluoromethanesulfonyl)imide ([AEMM]Am-TFSI)* (**1**): Yield 88%; ^1^H-NMR δ 1.25 (t, *J* = 7.3, 3H), 2.95 (s, 6H), 3.29 (q, *J* = 7.3, 2H), 3.91 (d, *J* = 7.1, 2H), 5.62 (d, *J* = 14.6, 1H), 5.62 (d, *J* = 11.9, 1H), 5.97-6.10 (m, 1H); ^13^C-NMR δ 7.6, 49.0 (t, *J*_C-N_ = 4.3), 58.5 (t, *J*_C-N_ = 2.5), 64.5 (t, *J*_C-N_ = 2.5), 119.4 (q, *J*_C-F_ = 319.5), 125.8, 127.3; Anal. calcd. for C_9_H_16_F_6_N_2_O_4_S_2_: C 27.4, H 4.1, N 7.1; found: C 27.5, H 4.2, N 7.1.

*Allyldimethylpropylammonium bis(trifluoromethanesulfonyl)imide ([AMMP]Am-TFSI)* (**2**): Yield 79%; ^1^H-NMR δ 0.89 (t, *J* = 7.3, 3H), 1.63-1.77 (m, 2H), 2.97 (s, 6H), 3.12-3.20 (m, 2H), 3.92 (d, *J* = 7.3, 2H), 5.61 (d, *J* = 16.1, 1H), 5.62 (d, *J* = 10.4, 1H), 5.97-6.11 (m, 1H); ^13^C-NMR δ 10.5, 15.3, 49.6 (t, *J*_C-N_ = 3.7), 64.4 (t, *J*_C-N_ = 2.5), 65.3 (t, *J*_C-N_ = 2.5), 119.5 (q, *J*_C-F_ = 319.5), 125.9, 127.4; Anal. calcd. for C_10_H_18_F_6_N_2_O_4_S_2_: C 29.4, H 4.4, N 6.9; found: C 29.3, H 4.5, N 6.8.

*Diallyldimethylammonium bis(trifluoromethanesulfonyl)imide ([AAMM]Am-TFSI)* (**3**): Yield 81%; ^1^H- NMR δ 2.95 (s, 3H), 3.91 (d, *J* = 7.3, 2H), 5.63 (d, *J* = 17.4, 1H), 5.64 (d, *J* = 10.1, 1H), 5.98-6.14 (m, 1H); ^13^C-NMR δ 49.2 (t, *J*_C-N_ = 4.4), 65.1 (t, *J*_C-N_ = 3.1), 119.4 (q, *J*_C-F_ = 319.5), 125.7, 127.6; Anal. calcd. for C_10_H_16_F_6_N_2_O_4_S_2_: C 29.6, H 4.0, N 6.9; found: C 29.7, H 3.9, N 6.9.

*Allylbutyldimethylammonium bis(trifluoromethanesulfonyl)imide ([ABMM]Am-TFSI)* (**4**): Yield 55%; ^1^H-NMR δ 0.93 (t, *J* = 7.1, 3H), 1.30 (sext, *J* = 7.5, 2H), 1.61-1.72 (m, 2H), 2.97 (s, 6H), 3.18-3.23 (m, 2H), 3.92 (d, *J* = 7.3, 2H), 5.62 (d, *J* = 15.4, 1H), 5.62 (d, *J* = 11.6, 1H), 5.97-6.11 (m, 1H); ^13^C-NMR δ 13.5, 19.2, 23.6, 49.6 (t, *J*_C-N_ = 4.3), 62.8 (t, *J*_C-N_ = 2.5), 65.2 (t, *J*_C-N_ = 3.1), 119.5 (q, *J*_C-F_ = 319.6), 126.0, 127.4; Anal. calcd. for C_11_H_20_F_6_N_2_O_4_S_2_: C 31.3, H 4.8, N 6.6; found: C 31.3, H 4.6, N 6.6.

*Allyldimethylhomoallylammonium bis(trifluoromethanesulfonyl)imide ([A(Ha)MM]Am-TFSI)* (**5**): Yield 64%; ^1^H-NMR δ 2.47-2.54 (m, 2H), 3.00 (s, 6H), 3.26-3.30 (m, 2H), 3.96 (d, *J* = 7.1, 2H), 5.15 (dd, *J* = 10.2 and 1.5, 1H), 5.22 (dd, *J* = 17.2 and 1.4, 1H), 5.63 (d, *J* = 14.6, 1H), 5.63 (d, *J* = 11.6, 1H), 5.68-5.81 (m, 1H), 5.98-6.12 (m, 1H); ^13^C-NMR δ 26.2, 49.6 (t, *J*_C-N_ = 3.8), 61.7 (t, *J*_C-N_ = 3.1), 65.3 (t, *J*_C-N_ = 2.5), 118.3, 119.4 (q, *J*_C-F_ = 319.6), 125.8, 127.5, 132.8; Anal. calcd. for C_11_H_18_F_6_N_2_O_4_S_2_: C 31.4, H 4.3, N 6.7; found: C 31.6, H 4.6, N 6.9.

*Allyldimethylmethoxymethylammonium bis(trifluoromethanesulfonyl)imide ([AMM(MOM)]Am-TFSI)* (**6**): Yield 81%; ^1^H-NMR δ 2.92 (s, 6H), 3.61 (s, 3H), 3.89 (d, *J* = 7.3, 2H), 4.58 (s, 2H), 5.61 (d, *J* = 17.8, 1H), 5.63 (d, *J* = 9.2, 1H), 5.94-6.08 (m, 1H); ^13^C-NMR δ 46.3 (t, *J*_C-N_ = 3.7), 60.7, 62.3, 91.1, 119.4 (q, *J*_C-F_ = 319.5), 125.4, 127.6; Anal. calcd. for C_9_H_16_F_6_N_2_O_5_S_2_: C 26.3, H 3.9, N 6.8; found: C 26.5, H 3.9, N 6.9.

*Allyldimethylethoxymethylammonium bis(trifluoromethanesulfonyl)imide ([A(EOM)MM]Am-TFSI)* (**7**): Yield 72%; ^1^H-NMR δ 1.21 (t, *J* = 7.0, 3H), 2.91 (s, 6H), 3.83 (q, *J* = 7.1, 2H), 3.88 (d, *J* = 7.3, 2H), 4.62 (s, 2H), 5.61 (d, *J* = 17.6, 1H), 5.62 (d, *J* = 9.9, 1H), 5.95-6.08 (m, 1H); ^13^C-NMR δ 14.9, 46.2 (t, *J*_C-N_ = 3.1), 62.3, 68.8, 89.7, 119.5 (q, *J*_C-F_ = 319.6), 125.4, 127.5; Anal. calcd. for C_10_H_18_F_6_N_2_O_5_S_2_: C 28.3, H 4.3, N 6.6; found: C 27.9, H 4.4, N 6.9.

*Allyldimethylmethylthiomethylammonium bis(trifluoromethanesulfonyl)imide ([AMM(MTM)]Am-TFSI)* (**8**): Yield 74%; ^1^H-NMR δ 2.38 (s, 3H), 2.97 (s, 6H), 3.96 (d, *J* = 7.3, 2H), 4.63 (s, 2H), 5.61 (d, *J* = 16.8, 1H), 5.64 (d, *J* = 9.4, 1H), 5.97-6.10 (m, 1H); ^13^C-NMR δ 17.9, 48.3 (t, *J*_C-N_ = 3.8), 64.5 (t, *J*_C-N_ = 3.1), 69.6, 119.4 (q, *J*_C-F_ = 319.5), 125.6, 127.7; Anal. calcd. for C_9_H_16_F_6_N_2_O_4_S_3_: C 25.4, H 3.8, N 6.6; found: C 25.2, H 4.0, N 6.7.

## Figures and Tables

**Table 1:** Physicochemical properties of the ILs prepared.

No	Ionic liquids	*T*_m_ (^o^C)	*T*_c_ (^o^C)	*T*_d_ (^o^C)	*η* (cP)	*σ* (mS/cm)
1	[AEMM]Am-TFSI (**1**)	13	-30	320	67	2.5
2	[AMMP]Am-TFSI (**2**)	2	-35	297	79	2.1
3	[AAMM]Am-TFSI (**3**)	-4	-44	294	66	3.2
4	[ABMM]Am-TFSI (**4**)	2	-58	297	113	2.5
5	[A(Ha)MM]Am-TFSI (**5**)	ND	ND	306	98	1.9
6	[AMM(MOM)]Am-TFSI (**6**)	ND	ND	268	47	3.9
7	[A(EOM)MM]Am-TFSI (**7**)	ND	ND	255	49	3.0
8	[AMM(MTM)]Am-TFSI (**8**)	ND	ND	229	161	1.1

ND: Not detected.

**Table 2:** Electrochemical windows of the ILs prepared ^a^.

No	Ionic liquids	Cathodic Limit (V)	Anodic Limit (V)
1	[AEMM]Am-TFSI (**1**)	-2.6	2.6
2	[AMMP]Am-TFSI (**2**)	-2.5	2.5
3	[AAMM]Am-TFSI (**3**)	-2.5	3.4
4	[ABMM]Am-TFSI (**4**)	-2.6	2.0
5	[A(Ha)MM]Am-TFSI (**5**)	-2.2	2.8
6	[AMM(MOM)]Am-TFSI (**6**)	-2.5	2.5
7	[A(EOM)MM]Am-TFSI (**7**)	-2.5	2.3
8	[AMM(MTM)]Am-TFSI (**8**)	-2.5	2.2

a. Working electrode: glassy carbon, counter electrode: Pt, reference electrode: Ag wire (calibrated with Fc/Fc^+^), scan rate: 10 mV s^-1^, cut-off current: 1 mA cm^-2^

**Table 3:** Electrochemical stability windows of the ILs with 1.0 M LiTFSI^a^.

No	Ionic liquids	Cathodic Limit (V)	Anodic Limit (V)
1	Carbonates^b^	0.66	4.76
2	[EM]Im-TFSI	0.75	5.21
3	[MMPP]Am-TFSI	0.66	5.78
4	[AMMP]Am-TFSI (**2**)	0.46	5.59
5	[AAMM]Am-TFSI (**3**)	0.51	6.43
6	[AMM(MOM)]Am-TFSI (**6**)	0.50	5.61

a. Working electrode: glassy carbon, counter electrode: Li, reference electrode: Li/Li^+^, scan rate: 5 mV s^-1^, cut-off current: 0.1 mA cm^-2^; b. 3:7 EC (ethylene carbonate):EMC (ethyl methyl carbonate), VC (vinylene carbonate, 3 wt%) with 1.3 M LiPF_6._

## Abbreviations

[EM]Im: 1-ethyl-3-methylimidazolium; [AM]Im: 1-allyl-3-methyl-imidazolium; [AA]Im: 1,3-diallylimidazolium; [MMPP]Am: dimethyldipropylammonium
